# Design, Optimization, Manufacture and Characterization of Efavirenz-Loaded Flaxseed Oil Nanoemulsions

**DOI:** 10.3390/pharmaceutics12090797

**Published:** 2020-08-23

**Authors:** Priveledge Mazonde, Sandile M. M. Khamanga, Roderick B. Walker

**Affiliations:** Department of Pharmaceutics, Faculty of Pharmacy, Rhodes University, Makhanda 6140, South Africa; pmazonde@live.com (P.M.); S.Khamanga@ru.ac.za (S.M.M.K.)

**Keywords:** nanoemulsion(s), phase-behavior, DoE, D-optimal design, vegetable oils, non-ionic surfactants, efavirenz, flaxseed oil

## Abstract

The formation, manufacture and characterization of low energy water-in-oil (w/o) nanoemulsions prepared using cold pressed flaxseed oil containing efavirenz was investigated. Pseudo-ternary phase diagrams were constructed to identify the nanoemulsion region(s). Other potential lipid-based drug delivery phases containing flaxseed oil with 1:1 *m/m* surfactant mixture of Tween^®^ 80, Span^®^ 20 and different amounts of ethanol were tested to characterize the impact of surfactant mixture on emulsion formation. Flaxseed oil was used as the oil phase as efavirenz exhibited high solubility in the vehicle when compared to other vegetable oils tested. Optimization of surfactant mixtures was undertaken using design of experiments, specifically a D-optimal design with the flaxseed oil content set at 10% *m/m*. Two solutions from the desired optimization function were produced based on desirability and five nanoemulsion formulations were produced and characterized in terms of in vitro release of efavirenz, physical and chemical stability. Metastable nanoemulsions containing 10% *m/m* flaxseed oil were successfully manufactured and significant isotropic gel (semisolid) and o/w emulsions were observed during phase behavior studies. Droplet sizes ranged between 156 and 225 nm, zeta potential between −24 and −41 mV and all formulations were found to be monodisperse with polydispersity indices ≤ 0.487.

## 1. Introduction

The human immunodeficiency virus (HIV) is a global health burden. At the end of June 2019, approximately 24.5 million people were accessing antiretroviral therapy [1]. In efforts to improve access to HIV drugs, simplification of process chemistry, reformulation, dose reduction, inclusion of new drug classes and new therapeutic strategies have been developed and contributed to the reduction of the HIV burden [2]. Efavirenz (EFV) is a non-nucleoside reverse-transcriptase inhibitor (NNRTI) used, in combination, for first line treatment of HIV. EFV is a BCS Class II or poorly soluble and highly permeable compound with an aqueous solubility of <10 mg/mL and low bioavailability of approximately 40% [3]. Caco-2 and intestinal permeability studies suggest EFV is highly permeable [4] and the clinical efficacy may be limited by low solubility. The log P of 4.6 for EFV suggests it may be a good candidate for formulation into a lipid based drug delivery system [5,6].

Lipid-based drug delivery systems (LBDDS) specifically, self-nanoemulsifying drug delivery systems (SNEDDS) are a useful approach for overcoming poor solubility and low oral bioavailability of some drugs [7,8,9]. The different classes of LBDDS include micro and nanoemulsions, of which the latter are single optically isotropic and metastable liquid solutions with droplet sizes ranging between 20 nm and 600 nm with some published reports suggesting a maximum particle size of 300 nm and 1000 nm [10,11,12]. Microemulsions are mixtures of oil, water and surfactant which are single optically isotropic, transparent thermodynamically stable solutions which form spontaneously when thermodynamic variables such as temperature and composition are met with droplet sizes of up to 100 nm [13].

Crude edible vegetable oils are readily available, economic and green chemistry products with functional food properties that exhibit numerous health benefits for patients and have been used in LBDDS [14]. Grapeseed, flaxseed and soybean oil are rich in poly unsaturated fatty acid, such as α-linoleic acid, content which is positively associated with cardiovascular health due to down-regulation of low-density lipoprotein cholesterol production. Diets rich in α-linoleic acid inhibit lymphocyte proliferation and the immune response in healthy humans and thus may be beneficial to individuals that present with autoimmune disorders [15]. Nanoemulsions that require low-energy input are relatively simple and inexpensive to manufacture if an appropriate surfactant mixture for a specific oil phase is used however, research relating to the formation, phase behavior and microstructure of nanoemulsions produced using food grade materials has not been conducted. Crude edible oils are difficult to solubilize in o/w nanoemulsions [16] and low-energy production methods take advantage of the intrinsic physicochemical properties of the components in order to generate sub-micron droplets. The use of a co-surfactant and/or co-solvent may be required to solubilize multicomponent oil or crude cold pressed materials sufficiently, to ensure that single or isotropic phases are formed. Ethanol used in concentrations ranging between 10% and 20% *v/v* is acceptable for food applications and can be used as a co-solvent to produce nanoemulsions. Legislation about the use of ethanol as an excipient, particularly for pediatric medicines differs from country to country, consequently formulations should comply with the regulatory requirements in the country of origin [17]. Co-solvents may improve the solubility of the oil, surfactant, co-surfactant and active pharmaceutical compounds while also functioning as penetration enhancers or altering bulk properties such as viscosity, density, refractive index and interfacial tension of aqueous solutions [18].

Nanoemulsions have small droplet sizes that ensure a large interfacial surface area is available to facilitate drug absorption and nanoemulsions or lipid based nanoformulations can enhance intestinal lymphatic transport of drugs and therefore circumvent the hepatic first pass effect [19]. Mechanisms of transport include an increase in membrane fluidity that facilitates transcellular absorption and opening of tight junctions that facilitate paracellular transport [20].

Critical quality attributes (CQA) of nanoemulsions include droplet size (PS), polydispersity index (PDI), zeta potential (ZP) and drug loading capacity (DLC). In addition, there is a need to understand physiological processes such as hepatic uptake and accumulation, tissue diffusion, tissue extravasation and renal excretion as a function of droplet size. Nano carriers of diameter between 100 and 150 nm circulating in blood vessels do not easily leave the capillaries that perfuse tissues such as the kidney, lung, heart and brain. In contrast smaller droplets between 20 and 100 nm may distribute into the bone marrow, spleen and liver sinusoids and may leave the vasculature via fenestrated capillaries in the perfused organs [21,22]. In the case of efavirenz and associated central nervous system adverse effects, small droplet sizes < 100 nm may lead to more pronounced side effects due to the associated increase in blood brain barrier permeability and the pathophysiology of acute and/or chronic CNS disease [23,24]. The PDI describes the uniformity or lack thereof of the size distribution of particles and PDI values < 0.05 are only observed for monodisperse standards whereas PDI values > 0.7 indicate that the sample exhibits a broad size distribution. The ZP can influence the stability of products, cellular uptake and intracellular trafficking of emulsions [25]. Generally, nanoemulsions with a high positive or negative ZP are electrically stabilized while emulsions with a low ZP tend to coagulate or flocculate leading to poor physical stability. High positive values for ZP > 30 mV or negative values < −30 mV tend to exhibit enhanced physical stability. In contrast low values for ZP of <5 mV can lead to droplet agglomeration [26]. The optimization of mixtures to produce a product of predefined specifications through estimation of the effects of formulation components on the mixture can be achieved using design of experiments (DoE) such as box-Behnken or D-optimal mixture designs with the aid of response surface methodology (RSM). The D-optimal mixed design has been applied to product formulation in the food, pharmaceutical and cosmeceutical industries as a reduced number of experiments are used to generate data for which interactions between variables can be identified using statistical tools thereby avoiding the shortcomings of traditional “one factor at a time” experimental and/or manufacturing approaches [27,28,29].

## 2. Materials and Methods

Cold pressed flaxseed, soybean, sunflower, olive, grapeseed and macadamia oils were purchased from Escentia products (Johannesburg, Gauteng, South Africa). Span^®^ 20 and Tween^®^ 80 were purchased from Merck (Johannesburg, Gauteng, South Africa). HPLC grade acetonitrile from Burdick and Jackson™ and ethanol were purchased from Anatech (Olivedale, Gauteng, South Africa). EFV (Form 1) was donated by Adcock Ingram^®^ Limited (Wadeville, Gauteng, South Africa). HPLC-grade water was produced using a RephiLe Bioscience Direct-Pure^®^ Ultrapure RO Water system, (Boston, MA, USA). 600 mg EFV tablets produced by Adcock Ingram Limited (Midrand, South Africa), Cipla Medpro (Cape Town, South Africa), Aspen Pharmacare Limited (Port Elizabeth, Eastern Cape, South Africa) and Aurobindo Pharma Limited (Alberton, South Africa) were purchased from a local pharmacy. Unless otherwise indicated, all materials were at least of analytical grade and were used without further purification.

### 2.1. Quantitative Determination of Efavirenz

A reversed-phase high-performance liquid chromatographic (HPLC) method was developed and validated for the quantitation of EFV in solubility, loading capacity and in vitro release studies. The HPLC system consisted of a Waters^®^ Alliance e2695 (Waters^®^ Corporation, Milford, MA, USA) solvent delivery module, autosampler, an online degasser and a Waters^®^ 2489 dual wavelength UV-vis detector (Waters^®^ Corporation, Milford, MA, USA). The HPLC chromatographic separation was achieved using a Phenomenex Luna^®^ C18 (2) 100 A 150 mm × 4.6 mm i.d. stationary phase (Separations, Johannesburg, Gauteng, South Africa) and a mobile phase flow rate of 0.8 mL/min at a wavelength of 247 nm. The injection volume was 10 μL and mobile phase was 85:15 *v/v* acetonitrile and 0.015-M acetate buffer of pH 4.5. The method was linear over the concentration range 1–350 μg/mL with a R^2^ = 0.9958, precise with the % RSD < 0.79% for all samples tested. The LOQ was 0.15 µg/mL and LOD was 0.06 µg/mL.

### 2.2. Solubility

The solubility of EFV in different vegetable oils was determined by adding an excess amount of efavirenz to 5 mL of flaxseed, sunflower, soybean, macadamia, grapeseed and olive oil in Kimax^®^ test tubes with Teflon^®^-lined crew caps (DWK Life Sciences, Hattenbergstr, Mainz, Germany). The tubes were agitated with the aid of cylindrical BRAND^®^ (Wertheim, Germany) PTFE, length 5 mm, diameter 2-mm magnetic stirring bars at 100 rpm for 48 h at laboratory air-conditioned room temperature (22 ± 2 °C) using an FMH STR-MH magnetic stirring hot plate purchased from (Lasec^®^ Group, Cape Town, South Africa). The samples were removed and centrifuged using a Damon IEC HN-SII centrifuge (Thermo Scientific, Waltham, MA, USA) at 3000 rpm for 15 min after which a 500-µL aliquot of the supernatant was collected and added to 50 mL ethanol and water in a ratio of 3:2 ratio prior to filtration through a Millipore^®^ automation compatible 0.45-μm PVDF membrane syringe filter from (Merck Group, Darmstadt, Germany). The concentration of EFV in the oils was determined using the validated HPLC method described in Section 2.1.

### 2.3. Formulation Design and Optimization

#### 2.3.1. Pseudo-Ternary Phase Diagrams and Emulsion Classification

**Figure 1:**
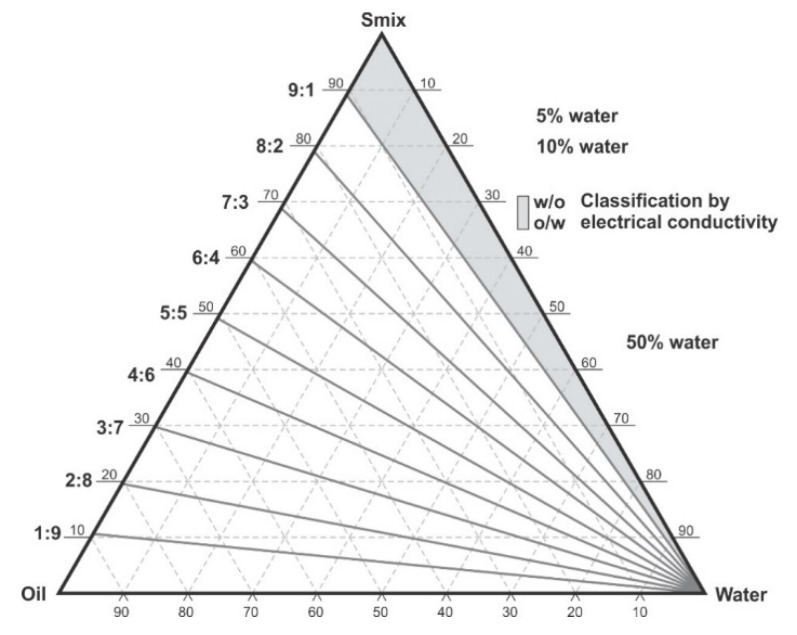
Pseudo-ternary phase diagram and scheme used for phase diagram plotting, showing dilution lines and areas in which electrical conductivity was tested. Adapted from [34].

**Figure 2:**
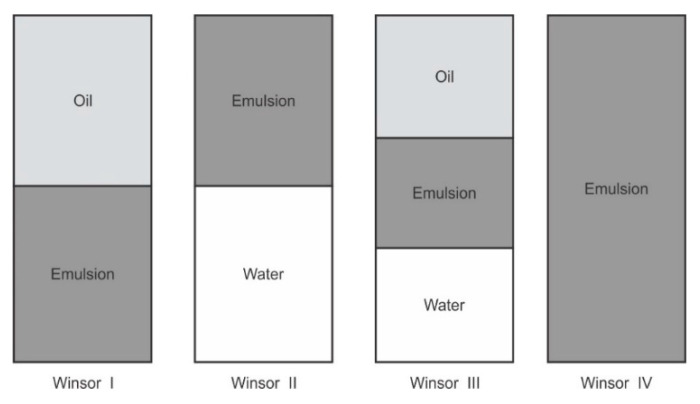
Winsor phase behavior used to assess phase diagram plots.

The water titration method was used to construct phase diagrams to identify the type of structure that resulted following emulsification and to characterize the behavior of mixtures along dilution paths [30]. Preliminary studies were performed making mixtures of flaxseed oil and a surfactant mixture of (Tween^®^ 80 and Span^®^ 20) (1:1) *m/m* in ratios of 9:1, 4:1, 7:3, 3:2, 1:1, 2:3, 3:7, 1:4 *m/m* and Winsor I-type products with no isotropic regions were produced at 22 ± 2 °C and observed after 48 h of incubation Therefore, a surfactant, co-surfactant and co-solvent mixture was considered for further phase behavior investigations. Surfactants solutions of Tween^®^ 80, Span^®^ 20 and ethanol were mixed using a Genie two vortex mixer (Scientific Industries, Inc.™, Bohemia, NY, USA) at 800 rpm for 30 ± 2 s. Three surfactant mixtures *viz*., S1, S2 and S3 comprised of combinations of ethanol: Tween^®^ 80: Span^®^ 20 in; 0.5:1:1, 1:1:1 and 1.5:1:1 *m/m* ratios, respectively. The surfactant mixtures were then added to flaxseed oil to produce pseudo-binary solutions in 9:1, 4:1, 7:3, 3:2, 1:1, 2:3, 3:7, 1:4, 1:9 *m/m* ratios to produce surfactant and oil mixtures in Kimax^®^ test-tubes (DWK Life Sciences, Hattenbergstr, Mainz, Germany). The pseudo-binary pre-concentrates mixtures contained no additional water. To minimize this effect, 500 mL of >98% ethanol was poured in a Schott Duran bottle (DWK Life Sciences, Hattenbergstr, Mainz, Germany) containing 300 g of 3A and 4A (1:1) *m/m* molecular sieve pellets from (B & M Scientific, Cape Town, South Africa), sealed and kept under air-conditioned laboratory room temperature for 7 days for further dehydration [31,32]. The ethanol was then degassed under vacuum with the aid of a Model A-2S Eyela aspirator degasser (Rikakikai Co., Ltd., Tokyo, Japan) and filtered through a 0.45-µm HVLP Durapore^®^ membrane filter (Millipore^®^ Corporation, Bedford, MA, USA) prior to use. Each of the ratios tested represent a dilution line from one to nine on the Gibbs phase triangle depicted in Figure 1. Water was added in 5% ± 1% increments to each pseudo-binary mixture following the titration chart summarized in Table 1 and after a 48-h incubation at room temperature, 22 ± 2 °C the regions of the phase diagram were identified and characterized for Winsor behavior visually prior to further characterization of pre-defined and identified formulation attributes. The titration chart and Gibbs triangle plots were developed using Triplot version 4.1.2 software (Todd A. Thompson, LA, USA). Points that were located within the phase diagram were observed and evaluated against the Winsor phase behavior descriptions [33] as depicted in Figure 2 and the data were plotted as a phase diagram, a graphical plot using Triplot software spreadsheet (titration chart), example is shown in Table 1. The resultant pseudo-ternary phase diagrams for surfactant-mixtures S1, S2 and S3 have been reported to show a representative sample of the types of structures that form when different amounts of ethanol as used and these data are shown in the results. The mixtures for the eight pseudo-binary solutions produced were vortexed using a Genie two vortex mixer (Scientific Industries, Inc.™, Bohemia, NY, USA) and placed into Falcon^®^ clear 24-well cell culture microplates with a lid from Corning^®,^ Inc (Corning, NY, USA). The bottom surface was placed onto a Xerox WorkCenter 3655 scanner (Xerox™, Norwalk, CT, USA) with the top surface lid and sides covered with a clean white background paper. The transparency and turbidity of the phase diagram dilution-line ratio mixtures were visualized by the scanner, characterized for droplet size, PDI and zeta potential. The images for each of the pseudo-binary solutions and corresponding droplet size, PDI and ZP elucidated immediately (within 6 min) after vortex mixing are listed in Table S1 in the Supplementary Material, for the of analyses performed in triplicate. Conductivity of points P1 to P20, in Table 1, which represent each 5% *w/w* increment point from 0% water to 95% water approaching the water vertex along dilution-line 9 for surfactant-mixture 1 (S1) was measured using a FiveEasy™ F30 conductivity meter (Mettler Toledo, Greifensee, Switzerland). The electrical conductivity was used to classify the microstructure of the emulsions and establish if w/o or o/w emulsions had formed as o/w systems exhibit higher electrical conductivity than w/o emulsions and these data are reported in Table 1.

#### 2.3.2. D-Optimal Design and Statistical Optimization

Surfactant-mixture optimization studies were performed using a D-optimal design to elucidate the effect(s) of the proportion of individual components of the surfactant mixture viz. Span^®^ 20, Tween^®^ 80 and ethanol content on droplet size, PDI and ZP. The D-optimal design studies were performed with the aid of Design-Expert version 12.0 software (Stat-Ease, Inc., Minneapolis, MN, USA). The proportion of oil in the nanoemulsion was maintained at 10% *v/v* for dilution-line 9, which falls in the nanoemulsion region. Span^®^ 20, Tween^®^ 80 and ethanol were independent variables and droplet size, PDI and ZP were the responses monitored. The oral route of delivery is proposed for these nanoemulsions, therefore the proportion of ethanol used was maintained at the permissible levels for food content with the largest concentrations at 20% *m/m* [35,36,37]. Legislation relating to the use of ethanol as an excipient in pediatric medicines or drugs is different in different countries and the American Academy of Pediatrics recommends that ethanol content in pediatric drugs should not produce a blood concentration >25 mg/100 mL following administration of a single recommended therapeutic dose. The dose size of EFV is 200 mg for pediatric patients and 600 mg for adults and as a nanoemulsion the dose unit is relatively small and is not expected to produce blood ethanol levels above regulated limits [38]. The European Medicines Agency (EMA) recommends a daily limit of 260.5 mg/kg/day of ethanol [39]. The levels for Span^®^ 20 (A), Tween^®^ 80 (B) and ethanol (C) used in the mixture fell in the range between the minimum and maximum levels listed in Table 2 with the sum of the components A, B and C always totaling 100%.

### 2.4. Preparation of Nanoemulsions

**Figure 3:**
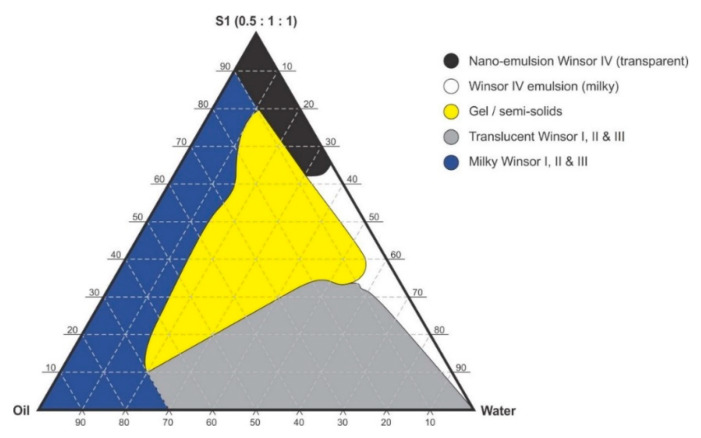
Pseudo-ternary phase diagram of flaxseed oil with surfactant-mixture S1 (0.5:1:1 *m/m* Ethanol: Tween^®^ 80: Span^®^ 20).

**Figure 4:**
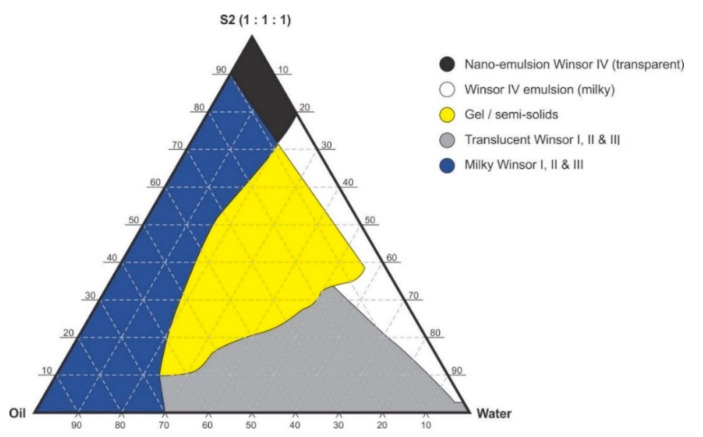
Pseudo-ternary phase diagram of flaxseed oil with surfactant-mixture S2 (1:1:1 *m/m* Ethanol: Tween^®^ 80: Span^®^ 20).

**Figure 5:**
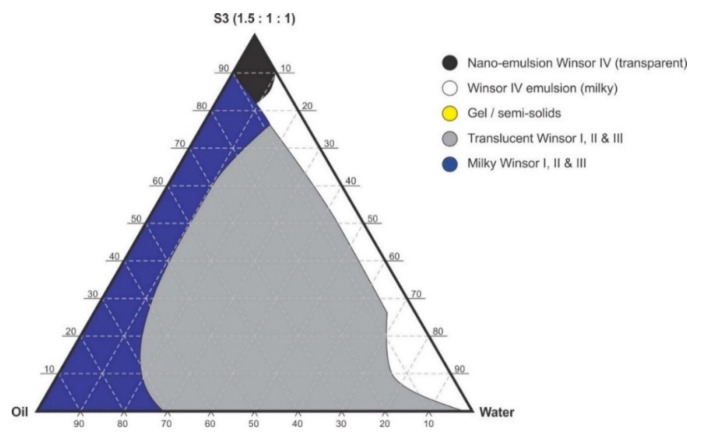
Pseudo-ternary phase diagram of flaxseed oil with surfactant-mixture S3 (1.5:1:1 *m/m* Ethanol: Tween^®^ 80: Span^®^ 20).

The nanoemulsion region was identified from the phase diagrams depicted in Figure 3, Figure 4 and Figure 5. Excess EFV was placed into a test tube containing the nanoemulsion preconcentrate that was one of five different surfactant mixtures and 10% *m/m* flaxseed oil. Of the five surfactant mixtures evaluated three were identified from each phase diagram and two were numeric optimization solutions extracted using the D-optimal design. The nanoemulsion mixtures were stirred at 100 rpm at laboratory room temperature under air-conditioner at 22 ± 2 °C with the aid of an FMH-STR magnetic stirrer (Lasec^®^, Cape Town, South Africa) for 48 h. The saturated nanoemulsions were centrifuged at 3000 rpm for 15 min using a model HN-SII IEC centrifuge (Thermo Scientific, Waltham, MA, USA) to separate excess drug from the nanoemulsion. Aliquots (500 µL) of the supernatant were dispersed in 50 mL water for droplet size and ZP measurements. A further 500-µL aliquot of the supernatant was mixed with 50 mL of a 3:2 ratio ethanol: water solution, prior to quantitation of EFV.

### 2.5. Characterization of Nanoemulsion Formulations

#### 2.5.1. Zeta Potential

The ZP was determined using a Nano-ZS Malvern Zetasizer (Malvern Instruments, Worchester, UK). A folded capillary was used for zeta-potential determinations with the instrument set in the Laser Doppler Anemometry (LDA) mode. The sample was prepared by dispersing 500-µL aliquots of the saturated nanoemulsion in 50 mL HPLC grade water and placed into a folded capillary cell. All measurements (*n* = 3) were performed at an applied field strength of 20 V/cm and the Helmholtz-Smoluchowski equation used to calculate the ZP of each sample, in situ.

#### 2.5.2. Polydispersity Index (PDI) and Droplet Size (PS)

The PDI and droplet size were determined using a Nano-ZS Malvern Zetasizer (Malvern Instruments, Worchester, UK) using the dynamic light scattering (DLS) mode. The sample was prepared by dispersing 500-µL aliquots of the saturated nanoemulsion in 50 mL HPLC grade water and placed into a 12.5 × 12.5 × 45 mm BRAND^®^ disposable cuvette (BRAND GmbH + CO KG, Wertheim, Germany).

#### 2.5.3. Transmission Electron Microscopy (TEM)

Transmission electron microscopy (TEM) was used to investigate the shape and surface morphology of the nanoemulsion and droplets in aqueous dispersions. Briefly, a drop of the aqueous nanoemulsion dispersion was placed onto a 3.05 mm copper grid fitted with a carbon film (FORMVAR/Carbon support 300 mesh) procured from TAAB Laboratories Equipment, Ltd., Alderson, Berks, RG7 8NA, UK. Excess liquid was removed using Whatman^®^ 110 hydrophilic filter paper (Whatman^®^ International, Ltd., Maidstone, UK) and the sample allowed to dry at room temperature (22 °C) for 24 h. The sample was visualized using a Zeiss^®^ Libra Model 120 TEM (Zeiss, GmbH, Germany) operating at an accelerating voltage of 80 kV.

#### 2.5.4. Raman Spectroscopy

Raman spectroscopy was used to identify all molecules present in the loaded and unloaded nanoemulsions to determine if new functional groups were evident sue to interaction of the components use in EFV loaded nanoemulsions. A Bruker Vertex 70-Ram II Raman spectrometer from (Bruker Optics, Inc., Billerica, MA, USA) equipped with a 1064 nm Nd: YAG laser for excitation in the region of 3400–80 cm^−1^ and liquid nitrogen cooled germanium detector was used to generate spectra that were acquired at 300 scans per minute. The instrument was set at 300 mW and the sample was placed in a hemispheric bore of an aluminum sample holder. The spectral resolution was 4 cm^−1^ and the spectra was processed using OPUS version 6.5 spectroscopy software (Bruker Optics, Inc., Billerica, MA, USA).

#### 2.5.5. Fourier-Transform Infrared Spectroscopy (FT-IR)

The presence of EFV in the nanoemulsions was also investigated using FT-IR and infrared absorption spectra were recorded using attenuated total reflection with a PerkinElmer spectrum 100 FT-IR spectrometer (PerkinElmer^®^ Pty, Ltd., Beaconsfield, UK) at 64 scans per minute over the frequency range 4000–650 cm^−1^. Nanoemulsion samples were mounted onto a diamond crystal using an applied force of approximately 100 N. The spectral data were processed using software FT-IR spectrum version 10.5.4 software (PerkinElmer^®^, Inc. Pty, Ltd., Beaconsfield, UK).

#### 2.5.6. In Vitro Release

A 300-µL aliquot of a saturated EFV nanoemulsion was placed into a size-00 hard gelatin capsule for in vitro EFV release studies. The dissolution time of the capsules (*n* = 6) in dissolution medium at 37 ± 1 °C was approximately 8 ± 1.03 min. In vitro dissolution studies were conducted using USP apparatus II with a Hanson Vision^®^ G2 Elite 8 dissolution bath fitted with a Vision AutoPlus and DissoScan™ auto sampler (Teledyne Hanson Research, Chatsworth, LA, USA) operated at a rotation speed of 50 rpm in 900 mL 0.1-M hydrochloric acid pH 1.2 with 1% *w/v* SLS at a temperature maintained at 37 ± 1 °C. A 10-mL sample was withdrawn at 15, 30, 60, 120, 390 and 720 min and filtered under vacuum with a Model A-2S Eyela aspirator degasser (Rikakikai Co., Ltd., Tokyo, Japan) through a 0.45-µm HVLP Durapore^®^ membrane filter (Millipore^®^ Corporation, Bedford, MA, USA) and the EFV released (*n* = 3) determined using the HPLC method described in 2.1. Sink conditions were maintained by replacement of fresh dissolution medium after removal of each sample.

## 3. Results

### 3.1. Solubility

The highest solubility of 89.41 mg/mL for EFV was observed for flaxseed oil and the solubility data are summarized in Table 3. Flaxseed oil was therefore used as the oil phase for constructing pseudo-ternary phase diagrams for the self-emulsifying nanoemulsions. In addition to the physicochemical properties of the EFV the molecular volume, polarity of the oil, chain length and saturation or unsaturation of triglyceride chains of the vegetable oils also influence solubility (MCT) have due to their higher fluidity, better solubility properties [40]. Medium chain triglycerides (MCT) containing oils are best for LBDDS as they are resistant to oxidation and possess high solvent capacity when compared to long-chain triglycerides (LCT) oils because of the high effective concentration of ester functional groups in the oil [41]. However, most of the vegetable oils selected for this study (Table 3) are predominantly and commonly composed of LCT and C_18_ chains. It was observed that as the proportion of the unsaturated component C_18:3_ component of the vegetable oils increases the solubility of EFV increased. Flaxseed oil has approximately 50% C_18:3_, soybean oil approximately 9.5% C_18:3_, grapeseed, sunflower and olive oil contain C_18:3_ of <2% [42,43,44].

### 3.2. Pseudo-Ternary Phase Diagrams

Pseudo-binary solutions of surfactant and oil in all ratios for the surfactant mixtures for dilution-line 1 to dilution-line 8 exhibited two phases of Winsor I-type phase behavior on standing for 30 min, but formed turbid mixtures following vortex mixing for 30 s at 800 rpm. Dilution-line 9 or ratio 9 resulted in a transparent and kinetically stable, isotropic system which exhibited Winsor IV-type behavior for up to 12 months at room temperature (22 °C). The droplet sizes of these ratio mixtures gradual increased as the proportion of flaxseed oil used increased while surfactant content decreased. The surfactant to oil ratio for the 9:1 pseudo-binary solution resulted in emulsions with the smallest droplet size and lowest PDI of all ratios tested. Both a nanoemulsion and microemulsion region was observed along dilution-line 9 for all three surfactant mixtures tested. Five distinct regions were formed viz., a nanoemulsion that is transparent Winsor IV, milky Winsor IV, i.e., cloudy isotropic mixture, translucent Winsor I, II and III, milky Winsor I, II and III in addition to a gel/semisolid region. Ratio 9 formed clear isotropic and transparent nanoemulsions with up to 35% *v/v* water addition for surfactant-mixture 1 as depicted in Figure 3, up to 20% *v/v* water for surfactant-mixture 2 as depicted in Figure 4 and only up to 5% *v/v* for surfactant-mixture 3 as depicted in Figure 5. While the nanoemulsion region area decreases as the ethanol content in the surfactant mixture increases, the milky isotropic and two or three-phase regions of the o/w emulsion also exhibit an increase in area whereas the gel region decreases in size with an increase in ethanol content, as the interfacial film is flexible, thereby solid structures are disrupted and the fluid phase areas increase in dimension. The dilute aqueous isotropic regions of cloudy o/w emulsions may be appropriate for an immediate release effect, due to the ease of dispersion of this phase in aqueous media [45]. Electrical conductivity measurements along dilution-line nine from the pseudo-binary solution of the surfactant/oil phase to the water vertex suggest that phase-inversion of system from a w/o to an o/w nano or microemulsion occurs at a specific point if not in a range of values as summarized in the titration chart followed in Table 1. The surfactant mixture for the S1 mixture exhibited the largest nanoemulsion region in the phase diagram and resulted in the production of the largest number of kinetically stable nanoemulsions following addition of water.

### 3.3. Statistical Analysis and Optimization

A single block D-optimal mixtures design was launched using the design constraints summarized in Table 2 on Design expert software version 12.0 software (Stat-Ease Inc., Minneapolis, MN, USA) to estimate the effects of the proportion of each of the surfactant-mixture components. To facilitate an understanding of the relationship(s) between one or more measured responses to input factors, a D-optimal design uses a sequential strategy that results in either first or second order polynomial mathematical relationships that can be best fit to statistical models such as quadratic, linear and cubic models for point prediction or optimization. The design produced was a simplex-lattice design with A + B + C = 1 or surfactant mixtures of 100% [46] which were then used for the manufacture of 10% *m/m* flaxseed oil containing nanoemulsions. A total of 16 runs with the compositions are summarized in Table 4 and the resulting droplet sizes, PDI and zeta potential. The most common empirical models fitted to experimental data include linear, quadratic or cubic models which increase in complexity of the polynomial from a 1st, 2nd, to 3rd degree, respectively. A 4th degree polynomial for systems involving composition, with the sum of the proportions by volume and weight has also been applied and reported [47]. Special quartic models are useful for modelling data generated from multicomponent mixtures and can be used to estimate multiple effects and the curvature of a response surface in the interior of a triangle to produce contour like effects [48,49]. All models were automatically fit by the design software for these data including linear, quadratic, cubic, special cubic, quartic and special quartic models were applied to the data then analyzed for the response variables monitored. The ANOVA analysis results are summarized in Table 5 for the suggested models and Table S2 in the Supplementary Material for all the models tested. The predicted residual sum of squares (PRESS) was used to establish the suitability of each model in respect of data fitting and the model with the lowest value for PRESS was identified as suitable for that response. The PRESS value is said to analyze the prediction ability of models and the model with the minimum PRESS is usually considered the best predictive model for a set of data [50]. ANOVA analysis following fitting of responses to each models are summarized in Table 5 to which droplet size and PDI were best fit to special quartic models while zeta potential was best fit to a linear model. Prior to predicting the optimized nanoemulsion formulation, composition residual analysis was undertaken to confirm that the assumptions for ANOVA analysis had been met. For this purpose, diagnostic plots viz., box Cox plots for residuals were plotted for all three responses and confirmed that data transformation was not required for droplet size and zeta potential and PDI. Diagnostic box Cox plots are depicted in Figures S3, S4 and S5 respectively in the Supplementary Material. When the statistical data are analyzed, input variables Span^®^ 20 (A) Tween^®^ 80 (B) and ethanol (C) are used to produce effects terms; A, B, C, AB, AC, BC, A^2^BC, AB^2^C, ABC^2^ which are tested to asses which ones are significantly different from 0, i.e., >0 therefore estimated to give coefficient values of correlation for prediction of a specific response. The largest positive coefficient of model terms represents the model term with the largest effect on a specific response.

#### 3.3.1. Droplet Size

**Figure 6:**
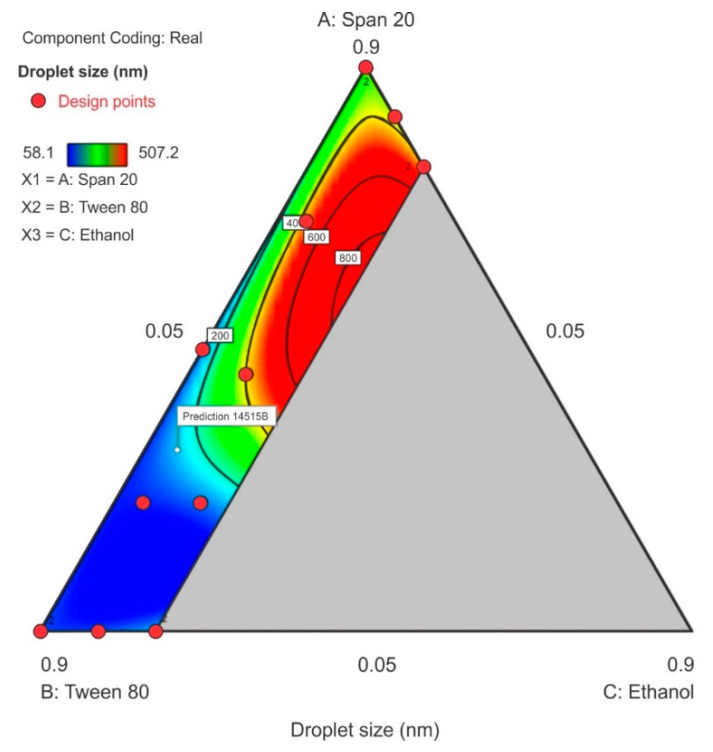
Contour plot for droplet size showing the characterized region where ethanol content is ≤20%.

The droplet size ranged from 58.1 nm to 507.2 nm with the smallest droplet sizes observed when Tween^®^ 80 was used at its maximum level of 0.9 in proportion in the surfactant-mixture design space whereas, the droplet size increased as Span^®^ 20 content increased. A sharp increase in particle size was observed as ethanol content is increased in the region of the Span^®^ 20 vertex as can be observed in the contour plot depicted in Figure 6. The model F-value of 8.91 indicated that the special quartic model for droplet size was significant and that there is only a 0.46% chance that a model F-value this large is due to noise. The *p*-value < 0.05 implied that the coefficients of the model terms were significantly different from zero, i.e., the effect of the model terms or combination of terms exerts an effect than can be estimated in the formulation composition. The model terms for the mixture A, B, C and A^2^BC were significant. The terms AB, AC, BC, AB^2^C and ABC^2^ were not significant and the ANOVA data and results are summarized in the supplementary data. The lack of fit F-value of 3.59 implied that lack of fit was not significant. The predicted R^2^ for droplet size was negative which implied that the mean data may be a better predictor for this response than using the model. However, adequate precision was >4 and was desirable showing that an adequate signal was observed and that the model could be used to navigate the design space and the model equation in terms of coded factors for droplet size is reported as Equation (1).

Droplet size = 342.00A + 90.75B + 3328.66C – 530.59AB–2941.83AC – 3530.63BC + 52,488.71A^2^BC – 11,742.82AB^2^C – 32,873.13ABC^2^ (1)

#### 3.3.2. Polydispersity Index

**Figure 7:**
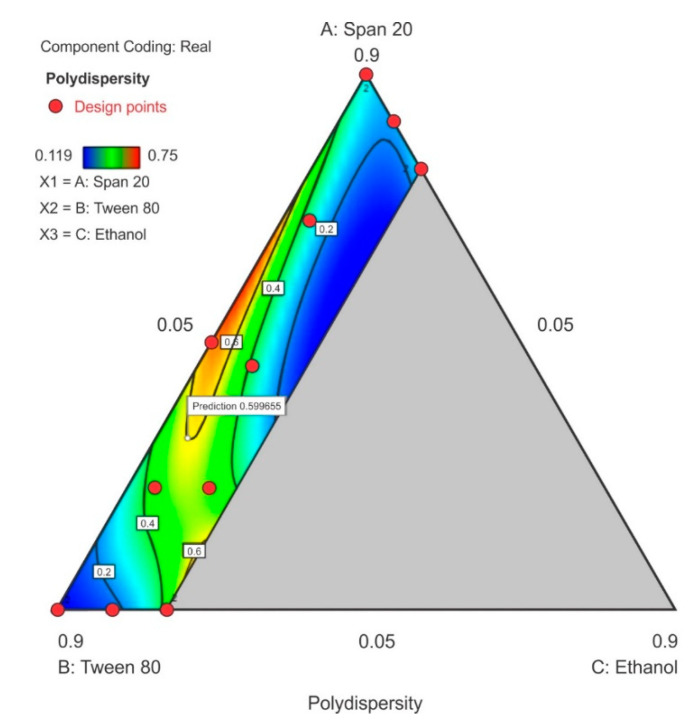
Contour plot for polydispersity index (PDI) showing point prediction when ethanol content is ≤20%.

The PDI ranged between 0.119 to 0.75 in the design space with the highest PDI observed at the center-point for Tween^®^ 80 and Span^®^ 20 content with ethanol content used closest to the lower limit. As the ethanol content was increased in the upper region of the contour plot towards the Span^®^ 20 vertex, a general decrease in PDI is observed as depicted in (Figure 7). The model F-value of 86.11 indicates that the special quartic model for PDI was significant and that there is only a 0.01% chance that a model F-value this large was due to noise. A *p*-value of <0.05 implied that the coefficients of effect and relationship of the model terms A, B, C, AB, AC, BC, A^2^BC, AB^2^C and ABC^2^ were significantly different from zero and could be estimated and the ANOVA data and results for this special quartic model are given in Table S4 of the Supplementary Material. The model equation in terms of coded factors for PDI is reported as Equation (2). The predicted R^2^ 0.8335 is in reasonable agreement with the adjusted R^2^ 0.9789. The lack of fit F-value 0.08 implies that lack of fit was not significant for the model and that there is a 56.02% chance that the lack of fit F-value this large is due to noise.

Polydispersity index = 0.2868A + 0.1155B + 10.85C + 2.19AB − 12.93AC − 10.78BC – 43.34A^2^BC + 1.016AB^2^C + 37.74ABC^2^ (2)

#### 3.3.3. Zeta Potential

**Figure 8:**
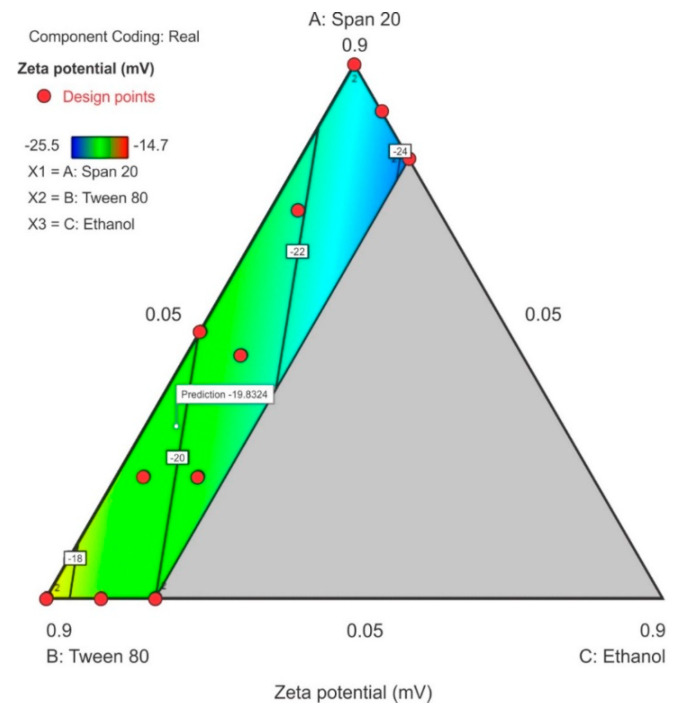
Contour plot for zeta potential (ZP) showing the point prediction when the ethanol content is ≤20%.

All components of the surfactant mixture exhibited a combined effect on the zeta potential and a linear relationship for this model is reported in Equation (3). The ethanol content exhibited the greatest effect on the zeta potential as observed by the magnitude of the coefficient for the term C and in the contour plot where 20% ethanol (C) was used. The contour plot for ZP is depicted in Figure 8 in which a significant region (in blue) revealed that as the concentration of Span^®^ 20 in the surfactant mixture was increased, the ZP decreased and became more negative with the lowest negative point occurring at the upper limit of ethanol and lower limit of Tween^®^ 80 content. The model F-value of 5.09 for ZP indicated that the linear model for ZP was significant and that there was only a 2.33% chance that a model F-value this large was due to noise. The probability *p*-value of < 0.05 implied the model terms were significant, the coefficients of the effect of each model term was significantly different from zero and could be estimated for this linear model, thus therefore only model terms A, B and C were significant within the formulation composition of these nanoemulsions. The equation in terms of coded factors for ZP is reported as equation 3. The predicted R^2^ of 0.1152 is not in close agreement with the adjusted R^2^ of 0.3530 suggesting a large block effect or possible problem with the model although, the adequate precision > 4 which is desirable and the adequate signal indicates that the model could be used to navigate the design space. The lack of fit F-value 0.49 implies that lack of fit was not significant for the model and there is an 82.64% chance that a lack of fit F-value this large is due to noise.

Zeta potential = −23.10A – 17.08B − 29.48C (3)

### 3.4. Optimization of Surfactant Mixtures and Assessment of Optimized Nanoemulsions

The optimization function was used to predict optimum levels for each for the components of the surfactant mixture. The primary criterion used was that the ethanol content should be minimized in the surfactant mixture. The second criterion required that a droplet size of between 100 and 200 nm was desired. Finally, the third and fourth criteria required minimization of the PDI and ZP. Two optimized solutions were produced based on the desirability function and the proportions of the formulation composition for the two solutions are reported as batches F4 and F5. Batches F1, F2 and F3 were nanoemulsion formulations made using arbitrary surfactant mixtures when assessing the phase behavior for the S1, S2 and S3 mixtures, respectively. The five formulations that were manufactured and assessed are listed in Table 6 with their respective compositions. The specified optimization criteria (constraints) in Table S6 and solutions produced are shown Table S7 with their associated desirability in the Supplementary Material. The prediction error was calculated against the experimental values obtained to give the prediction error of the D-optimal design. An overlay plot that depicts the area in which the desired optimization criteria is met is shown on Figure S6 in the Supplementary Materials.

### 3.5. Characterization and Assessment of Nanoemulsions

**Figure 9:**
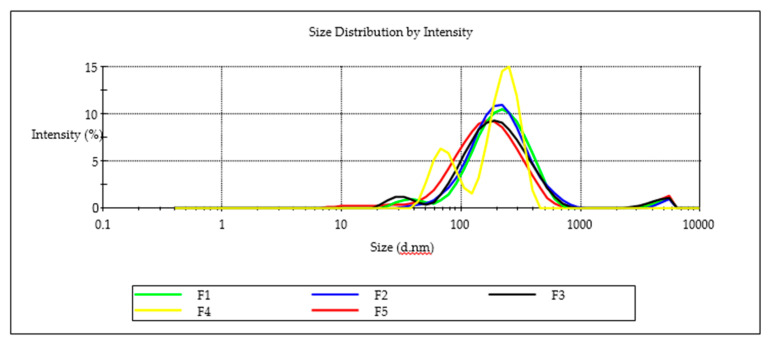
Particle-size distribution for batches F1, F2, F3, F4 and F5.

Nanoemulsion F3 was able to incorporate the largest amount of EFV of 571 mg/mL suggesting that the high ethanol content improved the solubility of EFV and miscibility of flaxseed oil, Tween^®^ 80 and Span^®^ 20 in the nanoemulsion. A decrease in EFV loading was observed as the proportion of ethanol in the composition was decreased. The highest PDI of 0.487, although arguably monodisperse for batch F4, is bimodal (Figure 9). The droplet size distribution of F4 could be attributed to the high Span^®^ 20 content due to a shift in HLB for these surfactant mixtures and the effect to thermodynamic stability [51]. A general decrease in the ZP was observed as the proportion of ethanol used was increased for batches F1 to F3 suggesting that the composition of F1, F2 and F3 would produce stable nanoemulsions over the long term. All five formulations exhibited ZP values < −20 mV suggesting the nanoemulsions that were produced were likely to be stable. The negative charge of nanoemulsion droplets may be useful for macrophage targeting since macrophages identify and take up negatively charged particles [52]. Macrophages are key factors in HIV infection and are significant cellular reservoirs of the HIV [53]. Emulsion droplets with a ZP of approximately ± 20 mV exhibit only short-term stability, with the tendency for the droplets to flocculate and coalesce [54]. To administer the recommended maximum adult dose of 600 mg formulation F3 would require a mass of 1.09 g of the nanoemulsion to be administered. The total mass is considerably lower and more convenient than the commercially available 600-mg EFV tablets manufactured by Cipla Medpro, Aurobindo, Adcock Ingram and Aspen Pharmacare for which tablets mass measured was 1.34 ± 0.09, 1.25 ± 0.11 g, 1.20 ± 0.08 g and 1.106 ± 0.045 g, respectively for (*n* = 20) suggesting that the nanoemulsion may produce a more convenient dosage form size for patients to use.

### 3.6. Transmission Electron Microscopy

**Figure 10:**
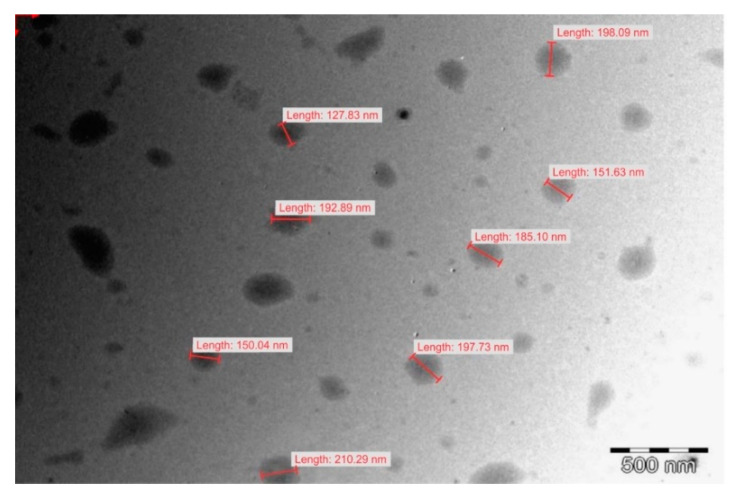
Transmission electron micrograph of nanoemulsion F5. The images for F1, F2, F3 and F4 are reported in the Supplementary Materials.

Transmission electron microscopy revealed the presence of largely spherical droplets of lipid as depicted in Figure 10 with an average droplet size in close agreement with that determined using dynamic light scattering. Smaller droplets in the size range 20–100 nm were present in the nanoemulsion dispersion in lower numbers as reflected in the droplet size distribution in Figure 8. These small droplet sizes may add to therapeutic possibilities by reducing the viral load in reservoirs associated with CNS tissue by distributing and perfusion into such tissues [55,56]. The bimodal distribution observed for droplet size distribution for F4 can be explained by the differences in the surfactant composition of the formulation. Ethanol exhibited the largest effect on solvent capacity and miscibility of the mixture for both surfactants, the oil phase and EFV. Rigid interfacial films give rise to bimodal distributions [57] and the greater the ethanol content, the more flexible the interfacial film of the immiscible phase. The bimodal distribution for F4 can be explained by the solvent capacity of the system for the lipophilic phase, Tween^®^ 80 and Span^®^ 20 which become miscible with addition of ethanol. As formulation F4 includes only a small proportion of ethanol, some proportion of the lipophilic phase (span^®^ 20 + 10% flaxseed oil) could possibly have been dispersed in agglomerates of a different size within the nanoemulsion mixture and larger than the small peaks of smaller particle sizes of F1, F2 and F3. Given the large concentration of ethanol in F3, the bimodal effects are seen to produce agglomerates within the nanoemulsion mixture of a smaller droplet size.

### 3.7. Raman Spectroscopy

The Raman spectra for EFV and EFV loaded nanoemulsions is reported in the Supplementary Materials (Figure S7). The EFV skeleton stretching vibrations were not affected by encapsulation of EFV in the nanoemulsion implying no interaction between EFV and lipids used in the formulation. The functional groups for EFV detected using Raman spectroscopy revealed that all expected signals observed for pure efavirenz and blank nanoemulsions were present and were in agreement with previously reported spectra [58]. The signal for the CH_2_ (A) functional group at 3093 cm^−1^, the C≡C (B) bond at 2250 cm^−1^, the (C=O) (C) bond at 1750 cm^−1^ and the C–H stretch at approximately 1000 cm^−1^ reflect the presence of EFV [59]. A comparison of experimentally determined vibrational wavenumbers for EFV is listed in the Supplementary Materials (Table S8).

### 3.8. Fourier-Transform Infrared Spectroscopy (FT-IR)

To understand the possibility of chemical interactions between the drug and nanoemulsion mixture, blank nanoemulsion, pure EFV, EFV loaded nanoemulsions and EFV nanoemulsions after dissolution testing were harvested left to dry in an open petri dish under room temperature over 72 h and characterized by FT-IR. The FT-IR spectrum of EFV loaded nanoemulsion in Figure S8 (in the Supplementary Material) showed the characteristic peaks of alkyne at 2247.63 cm^−1^, C–H stretch at 3000 cm^−1^, C–F stretch at 1400 cm^−1^, tertiary amide at 1602 cm^−1^ and C=O (D) at 1750 cm^−1^ [60]. A table of the comparison of the peaks reported in literature and those experimentally found are given in Table S8 in the Supplementary Material. The absorption band at approximately 3000 cm^−1^ was assigned to C–H stretching of the methylene group for Tween^®^ 80 and Span^®^ 20, the intensity of these peaks is diminished in the crystalline product harvested following dissolution testing and interaction of the EFV nanoemulsion with aqueous media resulting in conversion of electrons on carbon atoms changing from sp2 to sp3 [61]. The decrease in intensity of the O–H stretch detected as a broad band at 3500 cm^−1^ following dissolution testing may be due to the loss of ethanol from the nanoemulsion into the aqueous dissolution medium which results in an increase in the rate of nucleation and crystallization of EFV in solution.

### 3.9. In Vitro Efavirenz Release

**Figure 11:**
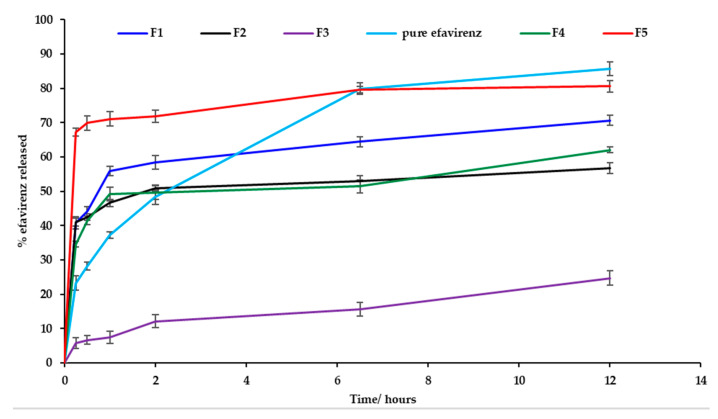
Dissolution profiles (mean ± SD (*n* = 3) for batches F1, F2, F3, F4 and F5 and EFV powder.

In vitro release testing revealed burst release of EFV for batches F1, F2, F4, F5 and for pure EFV within the first two hours of commencement of testing. Dissolution testing was conducted for the formulations listed in Table 6 and the % EFV released was based on actual drug loading data generated following assay of the nanoemulsion formulations. The nanoemulsion for batch F5 exhibited the greatest extent of release at 12 h that decreased for batches F1, F4, F5 and F3 which exhibited a sustained release effect and the release profiles are depicted in Figure 11. The reduction in amount of EFV released from the nanoemulsions may be attributed to solubilization of EFV in droplets of the nanoemulsion and/or formation of the EFV solvate on crystallization. Drugs may precipitate in vitro and in vivo due to a rapid change in pH, dilution with body fluids or digestion of solubilizing excipients subsequently resulting in lower EFV concentrations within the aqueous phase and better entrapment of EFV within the crystal lattice structure. As the saturation method of manufacture was used to produce the nanoemulsions, following supersaturation the process of nucleation continues and promotes crystallization [62,63]. As the proportion of ethanol is increased, the percent efavirenz released at 12 h decreases. On interaction with 1% *m/v* SLS in 0.1-M HCl dissolution fluid, the nanoemulsion formulations crystallized and formed a white edged crystalline semisolid that sank to the bottom of the dissolution vessel. Nanoemulsions of batch F4 and F5 were prepared with the same amount of ethanol and different proportions of Tween^®^ 80 and Span^®^ 20 and batch F5 released a larger amount of EFV than batch F4 at 12 h possibly due to the different hydrophilic lipophilic balance (HLB) value of the surfactants used. Tween^®^ 80 is soluble in an aqueous environment, whereas Span^®^ 20 is not and therefore Tween^®^ 80 interacts with the dissolution medium and contributed to better dissolution of EFV. Crystallization was observed for all batches and was greatest for batch F3. EFV precipitation is an undesirable outcome following administration of SNEDDS formulations. The loss of ethanol as observed with FT–IR analysis following dissolution testing may be the largest contributing factor to a reduced solubilization capacity and increased rate of nucleation in the supersaturated nanoemulsion formulations and hybridization of the C–H bond stretch that reduces the hydrophobicity of the formulation thereby affecting the interaction of EFV to form a solution.

## 4. Conclusions

The recommended dose of efavirenz for adults is 600 mg taken once daily and the most common dosage form available is a tablet, which is inconveniently large in size that may negatively affect patient adherence. The design of LBDDS focused on making the release characteristics independent of the gastrointestinal physiology and the fed/fasted state of the patient [64,65] and in a dosage form size that is small and convenient for the patient to use would be an advantage. The administration of EFV using these SNEDD technology would require a small unit size of the dosage form due to the high drug loading capacities exhibited which may produce a more convenient sized dosage form. Co-solvents and co-surfactants maybe used to improve the thermodynamic stability of formulations that exhibit an increased solubilization capacity resulting in enhanced therapeutic performance. The negative ZP of nanoemulsion droplets would be useful for macrophage targeting since macrophages identify and take up negatively charged particles [52]. Macrophages are key cells in HIV infection and are significant reservoirs of the virus [53].

Investigation of phase behavior of LBDDS components is useful for optimization of formulations and pre-formulation studies assist in defining appropriate proportions of each component to use, in addition to facilitation of decisions in relation to manufacturing processes such as whether high pressure or high shear homogenization can be used. Such decisions are required to ensure that an optimum product with predefined quality attributes is produced. The phase behavior of crude cold pressed flaxseed oil with non-ionic surfactants revealed an area within pseudo-ternary phase diagrams for surfactant-mixtures S1 and S2 that formed gels/semisolid structures which can be exploited for other drug delivery strategies such as topical application. This research identified that optimization of mixture compositions to produce a product with the required characteristics through estimation of the effects of the formulation components. However, accurate optimization using a D-optimal design may not be possible to only a relatively good predictability in droplet size alone and therefore other designs should be explored for future optimization.

Kinetically stable low energy nanoemulsions of flaxseed oil, Tween^®^ 80 and Span^®^ 20 surfactant and co-surfactant with ethanol were successfully manufactured. On visual observation, different release profiles for efavirenz were observed from different nanoemulsions. These can be exploited for further optimization to produce formulations suitable for undertaking in vivo and pharmacokinetic studies. The side effects of EFV associated with dose dumping may be reduced by using nanoemulsions to modulate release. The nanoemulsion approach is promising however, stability of formulations in gelatin or other encapsulated forms in which crystallization of EFV from solution is minimized, should be explored. The use of flaxseed oil in dosage forms intended for oral delivery may be beneficial to patients as there are health benefits associated with use of polyunsaturated fatty acids in addition to flaxseed oil being a cheap renewable raw material for dosage forms. Flaxseed oil contains an abundant source of, viscous fiber components and phytochemicals, such as lignans and protein that have demonstrated clinical activity as one of the six plant materials in the study of cancer-preventive foods [66].

## Figures and Tables

**Table 1:** Titration chart for use along each dilution line to plot phase diagrams with proportions of each component in the nanoemulsion with conductivity along dilution-line 9 for surfactant-mixture 1.

Water Addition Points on Dilution Line	Oil mg	S1 mg	Water µL	Total mg	S 1 %	Oil %	Water %	Conductivity µScm^−1^
P1	250	2250	0	2500	90.00	10.00	0.00	0.18
P2	250	2250	133	2633	85.45	9.49	5.05	0.2
P3	250	2250	280	2780	80.93	8.99	10.07	0.28
P4	250	2250	440	2940	76.53	8.50	14.96	0.45
P5	250	2250	625	3125	72.00	8.00	20.00	0.96
P6	250	2250	835	3335	67.46	7.49	25.03	1.67
P7	250	2250	1075	3575	62.93	6.99	30.06	7.76
P8	250	2250	1350	3850	58.44	6.49	35.06	11.43
P9	250	2250	1675	4175	53.89	5.98	40.11	25.3
P10	250	2250	2050	4550	49.45	5.49	45.05	119.6
P11	250	2250	2500	5000	45.00	5.00	50.00	142.2
P12	250	2250	3050	5550	40.54	4.504	54.95	147.6
P13	250	2250	3750	6250	36.00	4.00	60.00	173.6
P14	250	2250	4625	7125	31.57	3.50	64.91	173.9
P15	250	2250	5875	8375	26.86	2.98	70.14	202
P16	250	2250	7500	10,000	22.50	2.50	75.00	261
P17	250	2250	9950	12,450	18.07	2.00	79.91	278
P18	250	2250	14,275	16,775	13.41	1.49	85.09	356
P19	250	2250	22,500	25,000	9.00	1.00	90.00	389
P20	250	2250	46,600	49,100	4.58	0.50	94.90	433

**Table 2:** Constraints for input variables for D-optimal design.

Lower Limit %		Compound		Upper Limit %
5	≤	Span^®^ 20 (A)	≤	90
5	≤	Tween^®^ 80 (B)	≤	90
5	≤	Ethanol (C)	≤	20
		Total: A + B + C	=	100

**Table 3:** Saturation solubility of efavirenz (EFV) in vegetable oils.

Oil	Mean EFV Solubility (*n* = 3) ± SD mg/mL
Flaxseed	89.41 ± 0.12
Soybean	81.53 ± 0.18
Macadamia	71.31 ± 0.12
Grapeseed	69.83 ± 0.16
Olive	69.55 ± 0.09
Sunflower	55.99 ± 0.87

**Table 4:** Surfactant-mixture compositions generated by the D-optimal design and the experimental responses of 10% *m/m* flaxseed oil nanoemulsion in each run observed.

	Input Variables % *m/m*			Responses		
Run	Span^®^ 20 A	Tween^®^ 80 B	Ethanol C	Droplet Size nm	PDI	Zeta Potential mV
1	47.5	47.5	5	88.43	0.75	−23.85
2	66.875	24.375	8.75	408.25	0.332	−22.8
3	24.375	66.875	8.75	68.9	0.461	−21.9
4	5	82.5	12.5	173.4	0.176	−16.5
5	7.5	5	20	507.2	0.26	−23.2
6	7.5	5	20	404.4	0.324	−23
7	47.5	47.5	5	138.7	0.587	−18.8
8	5	75.0	20	180.56	0.481	−25.5
9	24.375	59.375	16.25	92.5	0.51	−18.6
10	5	90	5	70.5	0.12	−14.7
11	90.00	5	5	362.6	0.265	−21.4
12	5	75	20	58.1	0.412	−17.2
13	90	5	5	364.6	0.285	−21.9
14	43.75	43.75	12.5	441.1	0.365	−23.4
15	82.5	5	12.5	290.1	0.214	−23.4
16	5	90	5	70.75	0.119	−16.8

**Table 5:** ANOVA data for D-optimal responses and best-fit model.

Response	Predicted Model	f-Value	Degrees of Freedom	*p*-Value	R^2^	Adjusted R^2^	Predicted R^2^	Adequate Precision
Droplet size	Special quartic	8.91	8	0.0046	0.910	0.908	−1.618	7.205
Polydispersity	Special quartic	86.11	8	0.0001	0.9899	0.9789	0.8335	29.195
Zeta potential	Linear	5.09	2	0.0233	0.439	0.353	0.115	5.982

**Table 6:** Composition of the surfactant mixtures used for the manufacture of 10% flaxseed nanoemulsions and assessed in in vitro release and characterization studies.

Formulation	Span^®^ 20% *m/m*	Tween^®^ 80% *m/m*	Ethanol% *m/m*	Droplet Size nm	PDI	Zeta Potential mV	Efavirenz Content mg/mL	Mass of 1 mL of Nanoemulsion g
F1	40	40	20	185.1 ± 0.7	0.444 ± 0.003	−35.4 ± 0.9	377 ± 4.9	0.989 ± 0.006
F2	33.3	33.3	33.3	190.3 ± 2.0	0.387 ± 0.016	−34.4 ± 0.7	437 ± 13.1	1.040 ± 0.009
F3	28.5	28.5	42.8	156.8 ± 23.4	0.342 ± 0.048	−41.0 ± 0.9	571 ± 18.7	1.040 ± 0.012
F4	58.1	36.0	6.0	225.6 ± 16.8	0.487 ± 0.003	−31.9 ± 3.12	329 ± 9.45	0.817 ± 0.007
F5	32.2	58.3	9.5	146.7 ± 25.3	0.402 ± 0.012	−24.1 ± 2.33	334 ± 11.2	0.833 ± 0.009

## Supplementary Materials

The following are available online at https://www.mdpi.com/1999-4923/12/9/797/s1, Table S1: Scan images for investigation of turbidity and transparency during phase identification studies and results for selected critical quality attributes (CQA) viz., droplet size (DS), polydispersity index (PDI) and Zeta Potential (ZP), Table S2: PRESS statistic values and sum of squares for all models used to analyze the data, Table S3: ANOVA data for special quartic model for droplet size, df = degrees of freedom, Table S4: ANOVA data for special quartic model for polydispersity index, df = degrees of freedom, Table S5: ANOVA data for a linear model of Zeta Potential, df = degrees of freedom, Table S6: Constraints used for the target optimization criteria, Table S7: Solutions from Design Expert software for specified optimization criteria, Table S8: Theoretical (reported) and experimental wavenumbers characterized by FT-IR and Raman spectroscopy of efavirenz. Figure S1: Transmission electron micrograph of nanoemulsion F2, Figure S2: Transmission electron micrograph of nanoemulsion F4, Figure S3: (A) Diagnostic Box-Cox plot for power transforms and (B) predicted vs. actual plot for droplet size, Figure S4: (A) Diagnostic Box-Cox plot for power transforms and (B) predicted vs. actual plot for polydispersity index, Figure S5: (A) Diagnostic Box-Cox plot for power transforms and (B) predicted vs. actual plot for Zeta Potential, Figure S6: Overlay plot of the desirable area (yellow) derived using the specified criteria listed in Table S6, Figure S7: Raman Spectra of pure EFV, the control and EFV loaded nanoemulsions, Figure S8: FT-IR spectra of, pure EFV, Tween^®^ 80, Span^®^ 20, the control nanoemulsion, EFV loaded nanoemulsion and the nanoemulsion harvested following dissolution studies.
