# Supplementary material for: Design, Optimization, Manufacture and Characterization of Efavirenz-Loaded Flaxseed Oil Nanoemulsions
Source: Pharmaceutics. 2020 Aug 23;12(9):797. doi: 10.3390/pharmaceutics12090797 (PMC7559825; doi:10.3390/pharmaceutics12090797)
Supplement: Supplementary file 1 [file pharmaceutics-12-00797-s001.pdf]

Design, Optimization, Manufacture and Characterization of Efavirenz-Loaded Flaxseed Oil Nanoemulsions

Priveledge Mazonde, Sandile M. M. Khamanga and Roderick B. Walker *

Department of Pharmaceutics, Faculty of Pharmacy, Rhodes University, Makhanda 6140, South Africa; pmazonde@live.com (P.M.); S.Khamanga@ru.ac.za (S.M.M.K.)

* Correspondence: r.b.walker@ru.ac.za Tel.: +27-46-603-8412

Received: 20 July 2020; Accepted: 13 August 2020; Published: date

Table S1. Scan images for investigation of turbidity and transparency during phase identification studies and results for selected critical quality attributes (CQA) viz., droplet size (DS), polydispersity index (PDI) and Zeta Potential (ZP).

Ratio S_{mix} : Oil m/m	S1	CQA	S2	CQA	S3	CQA
9:1	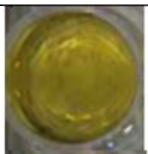	DS: 185.1 nm PDI: 0.408 Zeta: -35.4 mV	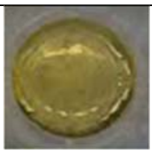	DS: 190.3 nm PDI: 0.207 Zeta: -34.4 mV	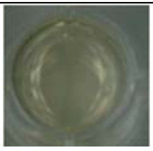	DS:156.8 nm PDI:0.266 Zeta:-43.9 mV
4:1	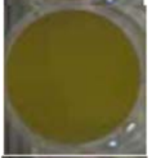	DS: 287.2 nm PDI: 0.407 Zeta: -35.4 mV	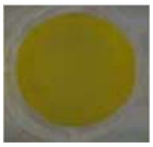	DS: 209.1 nm PDI: 0.305 Zeta: -35.3 mV	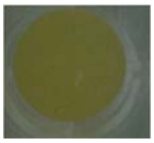	DS:519.7 nm PDI:0.307 Zeta:-31.0 mV
7:3	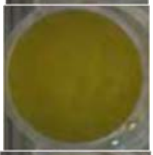	DS: 322.9 nm PDI: 0.412 Zeta:-40.3mV	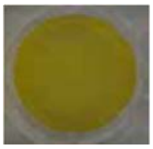	DS: 350.7 nm PDI: 0.72 Zeta: -29.9 mV	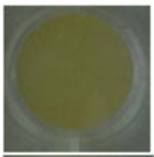	DS:1402 nm PDI:0.58 Zeta:-33.1 mV
3:2	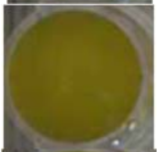	DS: 327.2 nm PDI: 0.277 Zeta: -34.9 mV	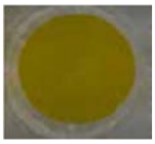	DS: 804.7 nm PDI: 0.682 Zeta: -33.5 mV	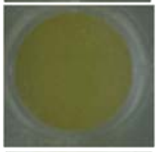	DS:829.7 nm PDI:0.26 Zeta:-41.9 mV
1:1	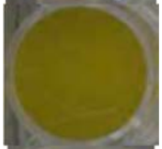	DS: 436.6 nm PDI: 0.214 Zeta: -35.0 mV	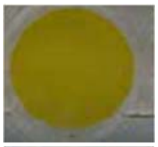	DS: 860.5 nm PDI: 0.782 Zeta: -32.2 mV	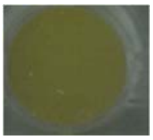	DS:844.3 nm PDI:0.38 Zeta:-43.8 mV
2:3	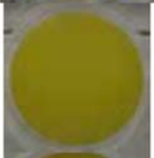	DS: 295.9 nm PDI: 0.316 Zeta: -45.8 mV	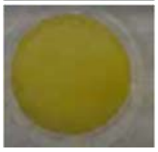	DS: 1441 nm PDI: 0.343 Zeta: -37.8 mV	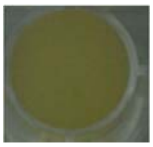	DS:774.7 nm PDI:0.22 Zeta:-40.7 mV
3:7	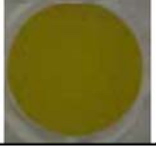	DS: 451.9 nm PDI: 0.363 Zeta: -41.0 mV	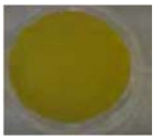	DS: 2380.7 nm PDI: 0.308 Zeta: -37 mV	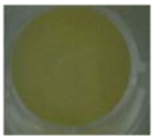	DS:667.2 nm PDI:0.69 Zeta:-40.4 mV

1:4	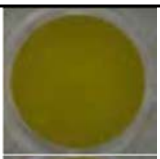	DS: 906.7 nm PDI: 0.236 Zeta: -47.8 mV	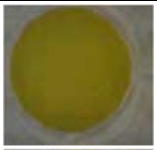	DS: 1617.7 nm PDI: 0.411 Zeta: -35 mV	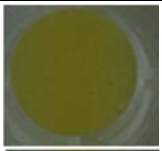	DS:1253 nm PDI:0.40 Zeta:-28.7 mV
1:9	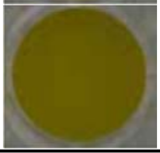	DS: 1149.0 nm PDI: 0.201 Zeta: -38.7 mV	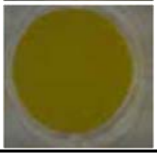	DS: 1023.1 nm PDI: 0.709 Zeta: -41.7 mV	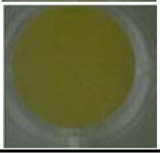	DS:1204 nm PDI:0.68 Zeta:-41.7 mV

Ratio 9:1 is seen to be clear and transparent, while the rest of the ratios are turbid on vortexing and Winsor type I products after standing for >30 minutes.

2. Transmission Electron Microscopy (TEM) Images.

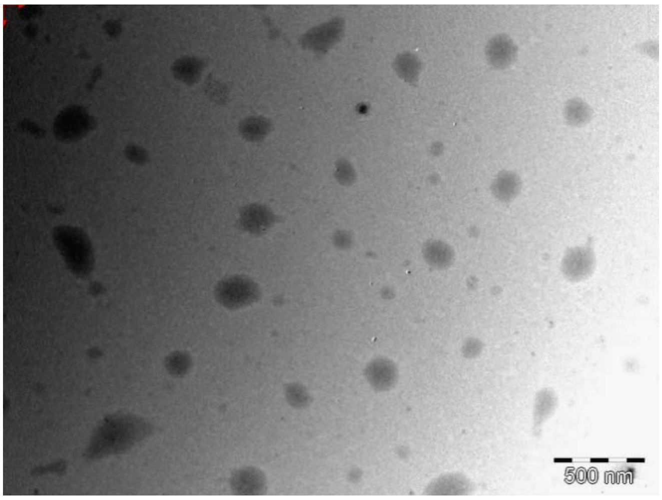

Figure S1. Transmission electron micrograph of nanoemulsion F2.

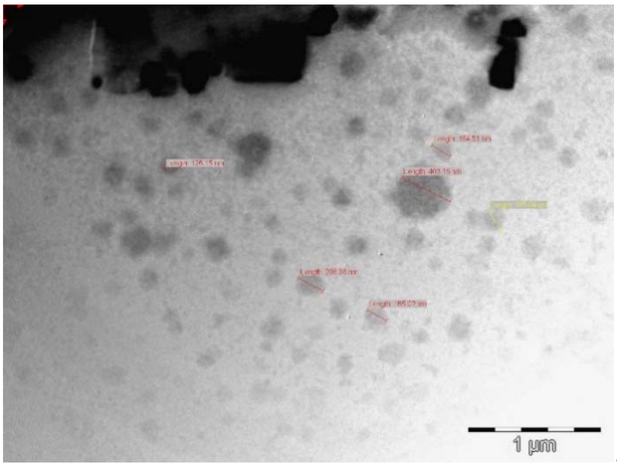

Figure S2. Transmission electron micrograph of nanoemulsion F4.

3. Statistical Analysis and Optimization.

Table S2. PRESS statistic values and sum of squares for all models used to analyze the data.

**Model**	**PRESS value for responses monitored**		
	**Droplet size**	**Polydispersity**	**Zeta Potential**
Linear	1.601E +05	5.43	138.58
Quadratic	2.480E +05	0.551	187.28
Cubic	6.933E +09	4612.21	1.349E +06
Special cubic	3.536E +05	0.5776	226.48
Special quartic	1.002E +06	0.2666	558.01
Sum of squares of suggested model	3.484E +05	3.97	68.8

The PRESS value is used to analyse the prediction ability of models and any model with a minimum value for the PRESS statistic is usually considered the most predictive model for a set of data. Following regression analysis, a check is required to establish if the model fits the data. Residuals may be used to establish if the model is unable to represent the data. Residuals are remainders of the response following fitting of data to a model (predictors) and are used to identify any unexplained patterns in the data when fitted to a model [1, 2]. Diagnostic Plots of residuals are depicted in Figures S3, S4 and S5.

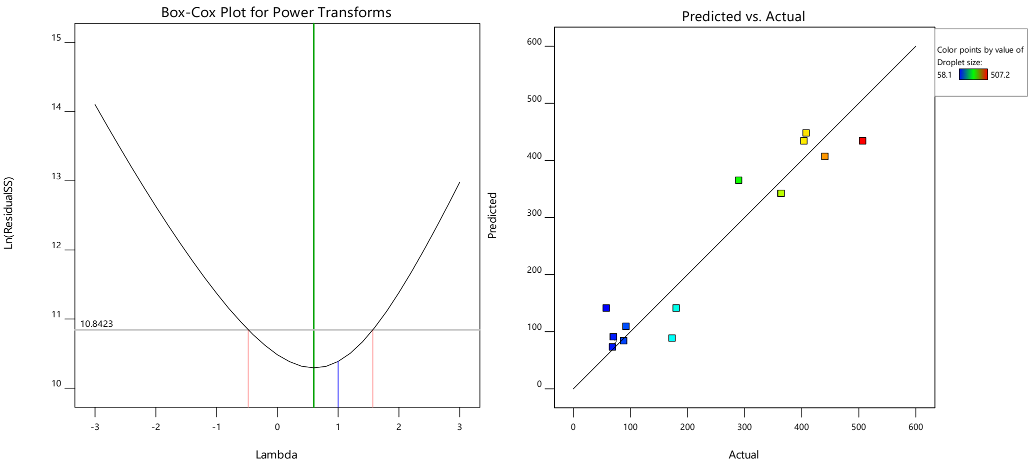

A B

Figure S3. (A) Diagnostic Box-Cox plot for power transforms and **(B)** predicted vs actual plot for droplet size.

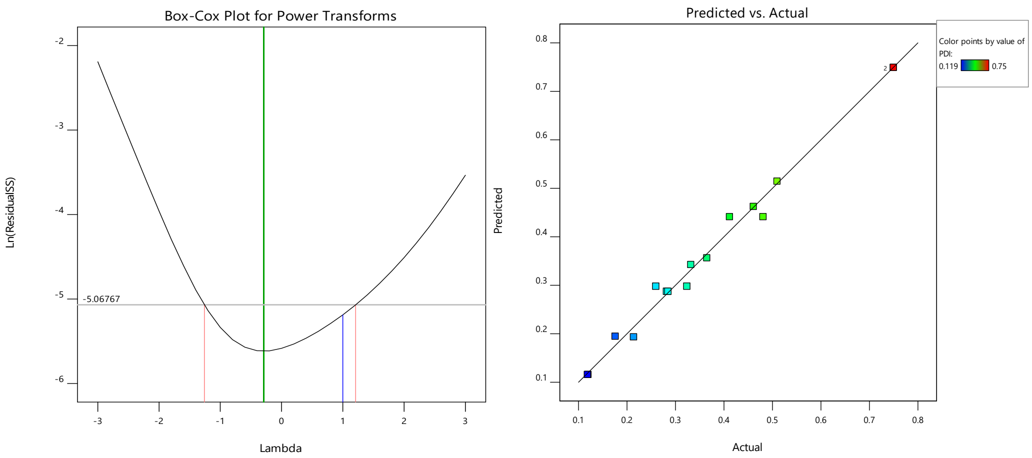

A B

Figure S4. (A) Diagnostic Box-Cox plot for power transforms and (B) predicted vs actual plot for polydispersity index.

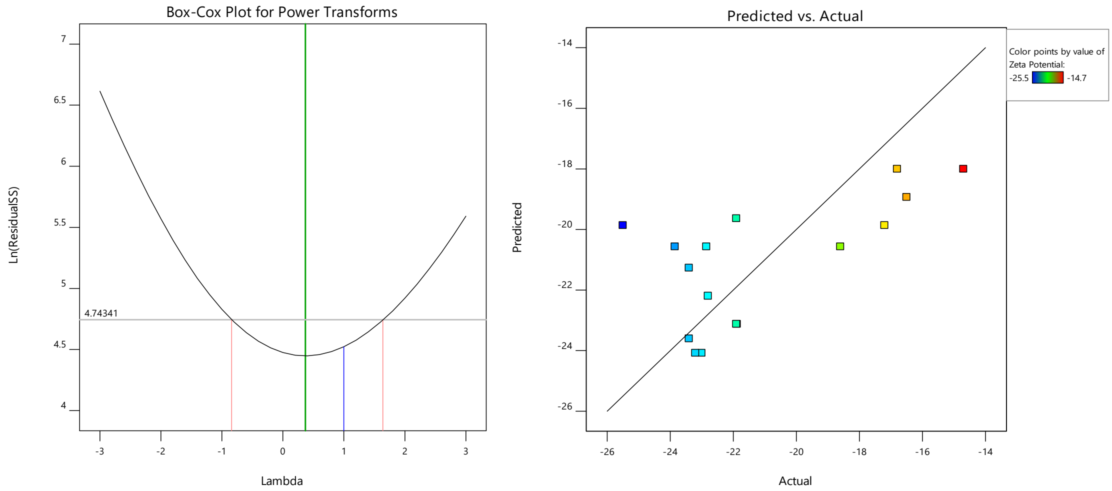

A B

Figure S5. (A) Diagnostic Box-Cox plot for power transforms and **(B)** predicted vs actual plot for Zeta Potential.

From the predicted *versus* actual scatter plots depicted in Figures S3 (B), S4 (B) and S5 (B), it is evident that not all data points fall exactly on the estimated regression line which means for a given set of input factors and conditions used there may be differences between the responses monitored and the predicted values. The difference or residual error is represented by parallel lines between the point of interest and the trend line [2]. Predicted vs actual scatter plots for Zeta Potential reveal a greater scatter than the other responses monitored which is reflected as unpredictability of the model for Zeta potential in this design space. It is clear that no distinctive pattern is evident in the plot for Zeta Potential whereas a linear pattern is evident for polydispersity index and droplet size.

Table S3. ANOVA data for special quartic model for droplet size, df = degrees of freedom.

Source	Sum of Squares	df	Mean Square	F-value	p-value	
**Model**	3.618E+05	8	45222.05	8.91	0.0046	significant
(1) Linear Mixture	2.675E+05	2	1.337E+05	28.89	0.0004	significant
AB	23590.52	1	23590.52	5.10	0.0586	
AC	845.21	1	845.21	0.1825	0.6820	
BC	1219.23	1	1219.23	0.2633	0.6236	
A^2BC	75719.26	1	75719.26	16.35	0.0049	significant
AB^2C	1434.95	1	1434.95	0.3099	0.5951	
ABC^2	457.54	1	457.54	0.0988	0.7624	
**Residual**	32410.38	7	4630.05			
Lack of Fit	19627.96	2	9813.98	3.59	0.0977	not significant
Pure Error	12782.42	5	2556.48			
**Cor Total**	3.942E+05	15				

Table S4. ANOVA data for special quartic model for polydispersity index, df = degrees of freedom.

Source	Sum of Squares	df	Mean Square	F-value	p-value	
**Model**	0.5429	8	0.0679	86.11	< 0.0001	significant
(1)Linear Mixture	0.0004	2	0.0002	0.2235	0.8052	
AB	0.4020	1	0.4020	503.39	< 0.0001	
AC	0.0158	1	0.0158	19.84	0.0030	
BC	0.0114	1	0.0114	14.22	0.0070	significant
A^2BC	0.0510	1	0.0510	63.83	< 0.0001	
AB^2C	0.0000	1	0.0000	0.0133	0.9115	
ABC^2	0.0006	1	0.0006	0.7551	0.4137	
**Residual**	0.0056	7	0.0008			
Lack of Fit	0.0012	2	0.0006	0.6522	0.5602	not significant
Pure Error	0.0044	5	0.0009			
**Cor Total**	0.5485	15				

Table S5. ANOVA data for a linear model for Zeta Potential, df = degrees of freedom.

Source	Sum of Squares	df	Mean Square	F-value	p-value	
**Model**	63.33	2	31.66	5.09	0.0233	significant
(1)Linear Mixture	63.33	2	31.66	5.09	0.0233	
**Residual**	92.11	13	7.09			
Lack of Fit	54.93	8	6.87	0.9237	0.5628	not significant
Pure Error	37.17	5	7.43			
**Cor Total**	155.43	15				

Table S6. Constraints used for target optimization criteria.

Name	Goal	Lower Limit	Upper Limit	Lower Weight	Upper Weight	Importance
A:Span® 20	is in range	0.05	0.9	1	1	3
B:Tween® 80	is in range	0.05	0.9	1	1	3
C:Ethanol	minimize	0.05	0.2	1	1	3
Droplet size	is in range	100	200	1	1	3
Polydispersity index	minimize	0.119	0.75	1	1	3
Zeta Potential	minimize	-25.5	-14.7	1	1	3

Table S7. Solutions from Design Expert software for specified optimization criteria.

Number	Span® 20 m/m %	Tween® 80 m/m %	Ethanol m/m %	Droplet Size nm	PDI	Zeta Potential mV	Desirability	Prediction Error for Droplet Size
1/F4	58.1	36.0	6.0	198	0.641	-21.320	0.463	12 %
2/F5	32.2	58.3	9.5	159	0.484	-20.204	0.531	8.33 %

The prediction error for the D-optimum design for F4 and F5 was calculated as a percent the using the formula, [(experimental value–predicted value)/experimental value] ×100. The prediction error for droplet size was 12 % for F4 and 8.33% for F5 revealing that this experimental design was the best predictor for droplet size whereas the prediction error for the polydispersity index was 31.6 % for F4 and -16.94% for F5. The model was not able to predict the Zeta Potential with prediction errors of 249 % and 219% for F4 and F5 respectively. Clearly accurate optimization using a D-optimal design may not be possible in this case as relatively good predictability was only observed for droplet size. It is therefore necessary to evaluate alternate experimental designs for future optimization investigations.

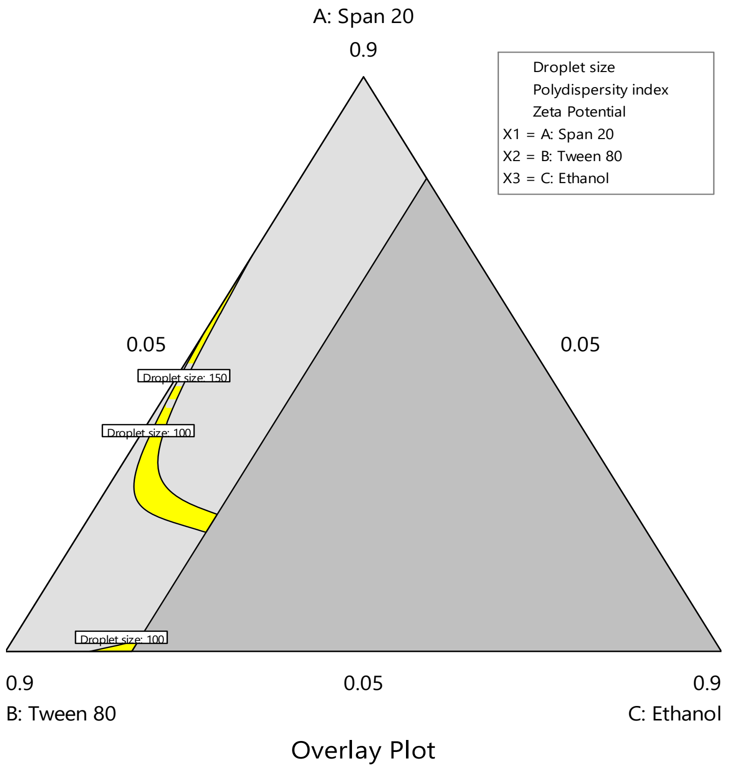

Figure S6. Overlay plot of the desirable area (yellow) derived using the specified criteria listed in Table S6.

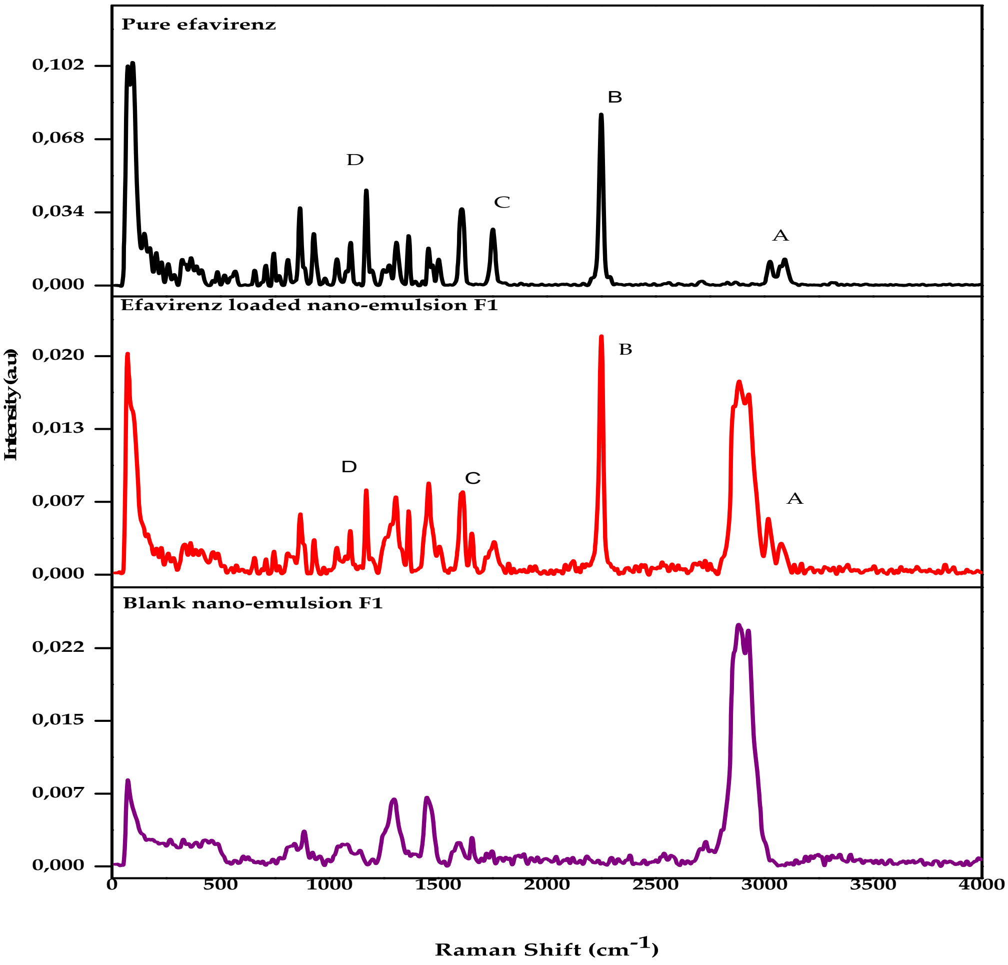

Figure S7. Raman Spectra of pure EFV, the control and EFV loaded nanoemulsions.

S8. Theoretical (reported) and experimental wavenumbers characterized by FT-IR and Raman spectroscopy of efavirenz.

**FT-IR Spectroscopy**		**Raman Spectroscopy**	
**Reported for Efavirenz cm^{-1} [3]**	**Observed cm^{-1}**	**Reported for Efavirenz cm^{-1} [4]**	**Observed cm^{-1}**
3097	-	3093	3090
3056	-	3071	3072
3020	-	3027	3022
2250	2247	2250	2250
1747	1750	1752	1751
1602	1600	1615	1614
1495	1500	1602	1606
1454	-	1500	1504
1429	1427	1472	-
1396	1400	1455	1454
1362	1362	1430	-
1317	1318	1395	-
1274	1272	1364	1367
1263	1260	1315	-
1242	1241	1306	1307
1198	1196	1275	-
1039	1037	1170	1170
978	976	1098	1099
923	919	1032	1039
889	877	977	931
850	848	941	871
818	817	743	746
		485	486
		410	418
		338	345

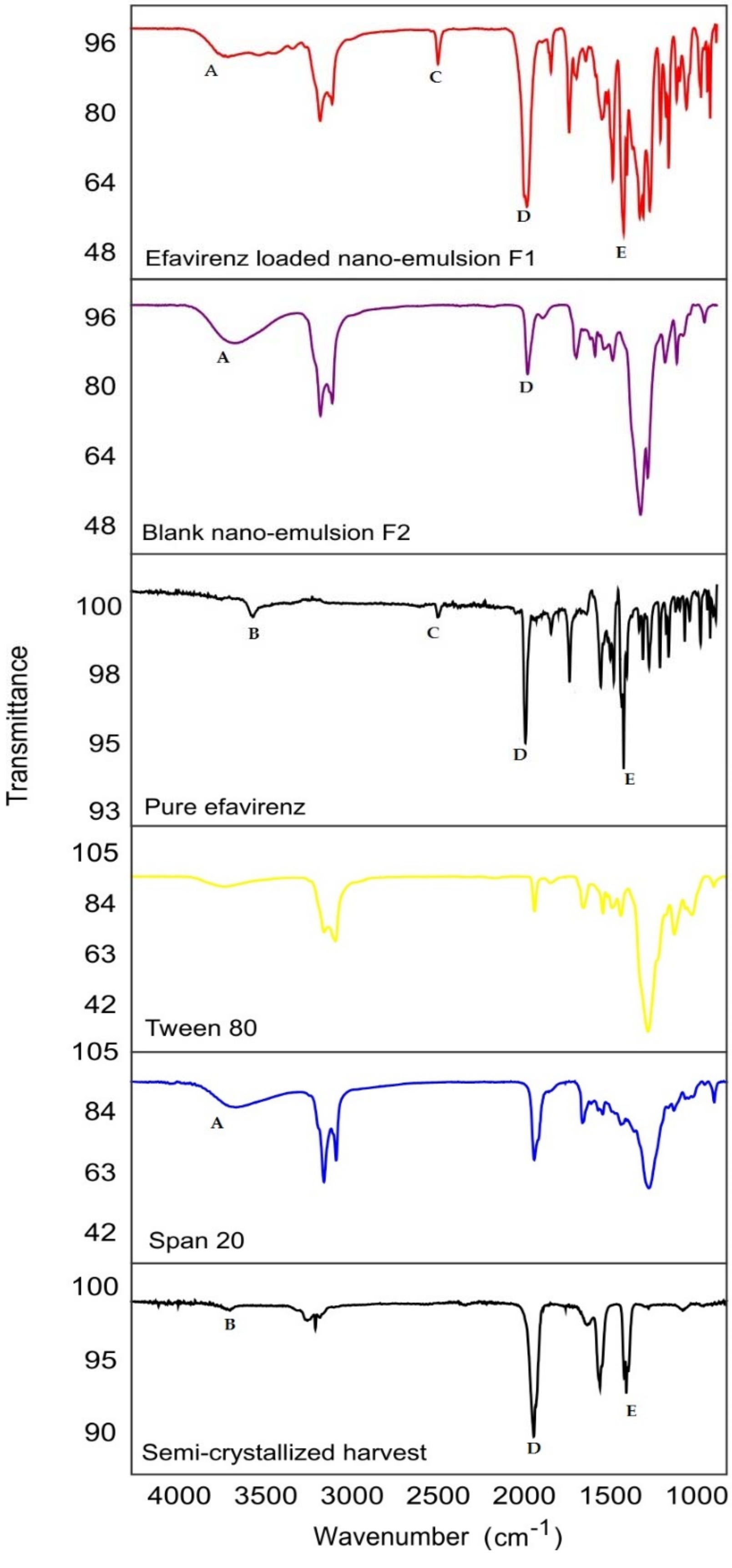

Figure S8. FTIR spectra of, pure EFV, Tween® 80, Span® 20, the control nanoemulsion, EFV loaded nanoemulsion and the nanoemulsion harvested following dissolution studies.

References

[1] N. I. Mohamad Zen, S. S. Abd Gani, R. Shamsudin, and H. R. Fard Masoumi, "The use of D-optimal mixture design in optimizing development of okara tablet formulation as a dietary supplement," *Sci. World J.*, vol. 2015, 2015, doi: 10.1155/2015/684319.

[2] U. S. D. of Commerce., *NIST/SEMATECH e-Handbook of Statistical Methods*. 2012. https://doi.org/10.18434/M32189

[3] N. P. Reddy, Y. Padmavathi, P. Mounika, and A. Anjali, "FTIR spectroscopy for estimation of efavirenz in raw material and tablet dosage form," *Int. Curr. Pharm. J.*, vol. 4, no. 6, pp. 390–395, 2015, doi: 10.3329/icpj.v4i6.23290.

[4] M. M. Marques *et al.*, "New solid forms of efavirenz: Synthesis, vibrational spectroscopy and quantum chemical calculations," *J. Mol. Struct.*, vol. 1137, pp. 476–484, Jun. 2017, doi: 10.1016/j.molstruc.2017.02.061.

© 2020 by the authors. Submitted for possible open access publication under the terms and conditions of the Creative Commons Attribution (CC BY) license (http://creativecommons.org/licenses/by/4.0/).
